# Novel associations between life’s crucial 9 components and cognitive health in a National Cohort of Older Americans

**DOI:** 10.1097/MD.0000000000046391

**Published:** 2026-05-12

**Authors:** Xiangyang Wang, Fan Wang, Weiwei Wang, Chaoshuai Hu, Junwei Wang, Hao Liu, Haigang Chang

**Affiliations:** a Xinxiang Medical University, Xinxiang, China.

**Keywords:** cognitive aging, life’s crucial 9, lifestyle factors, NHANES, older adults, public health prevention

## Abstract

This research sought to assess the relationship between LC9 and cognitive function among older adults in the United States. Data were sourced from the U.S. National Health and Nutrition Examination Survey 2011 to 2014. LC9 scores were derived from 9 health behaviors and factors. Cognitive function was evaluated using the CERAD Word Learning test, Animal Fluency test, and Digit Symbol Substitution Test. Multivariable linear regression models, restricted cubic spline analyses and subgroup analyses were utilized. Higher LC9 scores were significantly associated with better cognitive function across all models. In fully adjusted models, LC9 was positively associated with CERAD total score(β = 0.03, *P* = .006), delayed recall(β = 0.01, *P* = .024), Animal Fluency(β = 0.05, *P* = .012), and total cognitive function score(β = 0.19, *P* = .005). Among LC9 components, diet, blood pressure, blood glucose, and depression remained associated with cognitive function. The dose-response analysis supported these findings. Stronger associations were observed in participants ≥ 70 years and in women. This study shows a positive link between higher LC9 scores and cognitive performance in older U.S. adults. These findings have public health implications, lifestyle interventions could promote cognitive health and prevent decline.

## 1. Introduction

As the global population ages, cognitive decline and dementia have become major public health challenges worldwide. Currently, an estimated 55 million individuals live with dementia, a figure projected to rise to 150 million by 2050.^[1]^

This growing prevalence affects individuals and families and places substantial burdens on healthcare systems and long-term care infrastructures.^[2]^ Given the absence of curative treatments, identifying modifiable risk factors to preserve cognitive function and delay decline has become a central focus in aging and public health research.

In 2022, the American Heart Association introduced life’s essential 8 (LE8), a comprehensive framework for assessing cardiovascular health, which includes diet, physical activity, nicotine exposure, sleep health, body mass index (BMI), blood lipids, blood glucose (BG), and Blood pressure (BP).^[3]^ Building upon this framework, life’s crucial 9 (LC9), introduced in 2024, incorporates mental health as an essential domain.^[4]^ Notably, these lifestyle and cardiometabolic factors are increasingly recognized as shared determinants of cardiovascular and cognitive health, reinforcing the concept of convergence of heart-brain health throughout the life course. Emerging evidence suggests that a healthy diet,^[5]^ regular physical activity,^[6]^ and optimal sleep^[7]^ provide neuroprotective benefits, thereby supporting integrated prevention strategies that target multiple domains of aging-related health.

A growing body of evidence has established a positive association between higher LE8 scores and better cognitive outcomes. For instance, Zhu et al demonstrated that a higher LE8 score was significantly associated with superior performance in memory, executive function, and processing speed among older U.S. adults.^[8]^ Similarly, Liang and Zhang reported that both behavioral and biological components of LE8 were independently associated with reduced cognitive impairment, underscoring the role of comprehensive cardiovascular health in promoting cognitive aging.^[9]^ Beyond these studies, research by Christina Silvia Dintica et al further supports this link, showing that optimal cardiovascular health, as measured by LE8, is associated with a slower rate of cognitive decline in longitudinal assessments.^[10]^ The protective mechanisms are thought to be multifactorial, including enhanced cerebral blood flow, reduced neuroinflammation, and improved endothelial function facilitated by better cardiometabolic profiles.^[11,12]^

However, a critical limitation of the LE8 framework is its omission of psychological health. This is a significant gap given the well-established and strong association between mental health conditions (particularly depression) and cognitive decline.^[13–15]^ Depression has been linked to hyperactivity of the hypothalamic-pituitary-adrenal axis, increased inflammation, and hippocampal atrophy, all of which are pathways independent of, yet often comorbid with, cardiovascular risk factors.^[16,17]^ Therefore, an index that integrates both cardiovascular and psychological health may provide a more holistic and powerful assessment of an individual’s risk for cognitive impairment. While numerous studies have documented associations between LE8 and cognitive performance,^[8–12]^ the relationship between LC9 – a more comprehensive lifestyle and mental health index – and cognitive function remains underexplored. This study investigates the association between LC9 scores and cognitive outcomes in a nationally representative cohort of U.S. older adults, utilizing data from the National Health and Nutrition Examination Survey (NHANES) 2011 to 2014. We hypothesized that higher LC9 scores would be associated with better cognitive function. By generating robust epidemiologic evidence, this research seeks to inform public health strategies to promote cognitive resilience and prevent decline in aging populations.

## 2. Materials and methods

### 2.1. Study population

**Figure 1.**
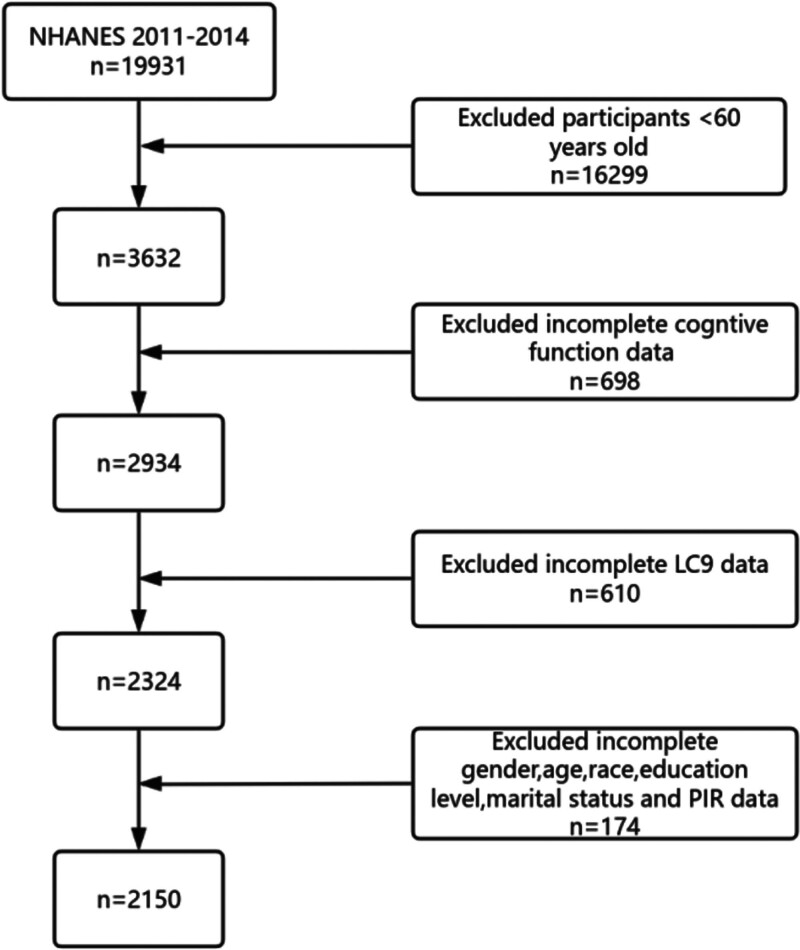
Flow diagram of participant selection for the analytic sample. A stepwise flow chart illustrating inclusion and exclusion criteria applied to the 2011 to 2014 NHANES dataset. Participants aged <60 yr, those with missing LC9 components, incomplete cognitive assessments, or missing covariates were excluded. The final analytic sample included 2150 participants. LC9 = life’s crucial 9, NHANES = National Health and Nutrition Examination Survey.

Data for this study were sourced from the NHANES, administered by the National Center for Health Statistics. NHANES utilizes a stratified, multistage, probability sampling design to produce nationally representative estimates of the non-institutionalized U.S. population. All participants provided written informed consent, and the National Center for Health Statistics Research Ethics Review Board approved the survey protocol. For this analysis, we employed data from 2 survey cycles (2011–2012 and 2013–2014), encompassing 19,931 participants. We selected data from 2 specific periods because cognitive assessments were fully available only during those periods. Additionally, we focused on individuals over 60 years of age, as the NHANES database conducted cognitive assessments exclusively for this age group. After excluding individuals younger than 60 years, those with missing covariate data, incomplete LC9 component data, or incomplete cognitive test data, the final analytic sample comprised 2150 participants (Fig. 1).

### 2.2. Cognitive function

Cognitive function was evaluated at NHANES Mobile Examination Centers utilizing 3 validated instruments: the CERAD word list learning (W-L) subtest, which assesses verbal memory through 3 immediate and 1 delayed recall trials. The scores for each trial range from 0 to 10, resulting in a total score of up to 30 for the immediate trials and up to 10 for delayed recall. The Animal Fluency Test, which measures categorical verbal fluency – a critical domain of executive function – yields scores ranging from 0 to 40. The digit symbol substitution test (DSST), a subtest from the Wechsler Adult Intelligence Scale, Third Edition (WAIS-III), evaluates processing speed, sustained attention, and working memory, with scores ranging from 0 to 100.

### 2.3. Measurement of life’s crucial 9

Life’s crucial 9 (LC9) represents a composite health metric that integrates 9 domains: 4 health behaviors (diet quality, physical activity, smoking status, and sleep health) and 5 health factors (body weight, blood lipids, BG, BP, and mental health). The scoring criteria for LC9 are delineated in Table S1, Supplemental Digital Content, https://links.lww.com/MD/Q844. Diet quality was evaluated using the 2015 Healthy Eating Index (HEI-2015), with the scoring procedures specified in Table S2, Supplemental Digital Content, https://links.lww.com/MD/Q844. Standardized NHANES questionnaires assessed mental health (patient health questionnaire-9), sleep duration, smoking status, and physical activity. Anthropometric measurements (height and weight) were obtained during clinical examinations, and BMI was calculated as weight in kilograms divided by height in meters squared (kg/m^2^). BP was measured following standardized protocols. Laboratory assays provided data for fasting BG, glycated hemoglobin (HbA1c), and non-HDL cholesterol concentrations.

### 2.4. Covariates

The covariates incorporated into the analysis were as follows: sex (male, female), age (treated as a continuous variable), race/ethnicity (Mexican American, Other Hispanic, Non-Hispanic White, Non-Hispanic Black, Other Race), educational attainment (categorized as less than high school, high school graduate, and greater than high school), marital status (married/living with partner, living alone), and family poverty-income ratio (PIR:<1.3, 1.3–3.5, ≥3.5).

### 2.5. Statistical analyses

All statistical analyses were performed utilizing R software (version 4.4.2) and Free Statistics software (version 1.9.2). Sampling weights (WTDR2D) were applied to account for the complex design of the NHANES survey, thereby ensuring nationally representative estimates. This weight is a composite weight specifically designed for analyzing the dietary recall interview data in NHANES. We reported both unstandardized regression coefficients (β) and standardized beta coefficients (β_std) derived from z-score transformed variables. The unstandardized coefficients indicate the absolute change in cognitive score per unit change in LC9 or its components, while standardized coefficients allow for comparison of effect sizes across different cognitive measures by expressing the change in terms of standard deviation units. Descriptive statistics for continuous variables were presented as means with standard deviations, and group comparisons were conducted using t-tests. Categorical variables were reported as weighted percentages, with comparisons using weighted chi-square tests. Associations between LC9 scores and cognitive function were assessed through multivariable linear regression models: Model 1 (unadjusted), Model 2 (adjusted for age), and Model 3 (fully adjusted for age, sex, race/ethnicity, education, marital status, and PIR). Associations between individual LC9 components and cognitive outcomes were also investigated. Restricted cubic spline models were employed to explore potential nonlinear associations. Subgroup analyses were conducted based on age group, sex, and income strata. Statistical significance was defined as *P* <.05.

## 3. Results

### 3.1. Baseline characteristics

Table 1 delineates the baseline characteristics of the study cohort. The mean age of the 2150 participants was 69.4 ± 6.8 years. The sample comprised 51.35% women and 48.65% men. Participants with elevated LC9 scores were more likely to be married or cohabiting, possess higher educational attainment, exhibit a higher PIR, and be more frequently identified as non-Hispanic White.

**Table 1:** Baseline characteristics of the study population, stratified by LC9 scores.

Characteristic	Level	Overall, N = 2150	Q1, N = 511	Q2, N = 558	Q3, N = 510	Q4, N = 571	*P*
Age (mean [SD])	–	69.414 (6.747)	68.358 (6.396)	69.520 (6.765)	69.875 (7.022)	69.846 (6.700)	.0003
Gender (%)	Male	1046.00 (48.65)	247.00 (48.34)	268.00 (48.03)	249.00 (48.82)	282.00 (49.39)	.9715
	Female	1104.00 (51.35)	264.00 (51.66)	290.00 (51.97)	261.00 (51.18)	289.00 (50.61)	–
Race (%)	Mexican American	178.00 (8.28)	46.00 (9.00)	56.00 (10.04)	42.00 (8.24)	34.00 (5.95)	<.0001
	Other Hispanic	199.00 (9.26)	53.00 (10.37)	56.00 (10.04)	48.00 (9.41)	42.00 (7.36)	–
	Non-Hispanic White	1118.00 (52.00)	211.00 (41.29)	260.00 (46.59)	289.00 (56.67)	358.00 (62.70)	–
	Non-Hispanic Black	483.00 (22.47)	179.00 (35.03)	133.00 (23.84)	90.00 (17.65)	81.00 (14.19)	–
	Other Race	172.00 (8.00)	22.00 (4.31)	53.00 (9.50)	41.00 (8.04)	56.00 (9.81)	–
Education level(%)	Under high school	201.00 (9.35)	74.00 (14.48)	58.00 (10.39)	36.00 (7.06)	33.00 (5.78)	<.0001
	High school	796.00 (37.02)	228.00 (44.62)	225.00 (40.32)	181.00 (35.49)	162.00 (28.37)	–
	Above high school	1153.00 (53.63)	209.00 (40.90)	275.00 (49.28)	293.00 (57.45)	376.00 (65.85)	–
Marital status (%)	Married or Living with partner	1271.00 (59.12)	262.00 (51.27)	333.00 (59.68)	302.00 (59.22)	374.00 (65.50)	<.0001
	Living alone	879.00 (40.88)	249.00 (48.73)	225.00 (40.32)	208.00 (40.78)	197.00 (34.50)	–
PIR (%)	<1.3	593.00 (27.58)	197.00 (38.55)	162.00 (29.03)	127.00 (24.90)	107.00 (18.74)	<.0001
	1.3–3.5	845.00 (39.30)	200.00 (39.14)	240.00 (43.01)	186.00 (36.47)	219.00 (38.35)	–
	≥3.5	712.00 (33.12)	114.00 (22.31)	156.00 (27.96)	197.00 (38.63)	245.00 (42.91)	–
CERAD: Total Score (3 Recall trials) (mean (SD))	–	19.179 (4.536)	18.746 (4.668)	19.113 (4.405)	19.327 (4.433)	19.497 (4.614)	.0499
CERAD: Delayed Recall Score (mean [SD])	–	6.072 (2.269)	5.912 (2.301)	6.088 (2.218)	6.094 (2.250)	6.180 (2.304)	.2821
Animal Fluency: Total Score(mean [SD])	–	16.992 (5.454)	16.080 (5.141)	16.672 (5.318)	17.169 (5.452)	17.961 (5.702)	<.0001
Digit Symbol: Score (mean [SD])	–	47.399 (16.777)	43.188 (16.000)	45.762 (17.122)	49.094 (16.555)	51.252 (16.276)	<.0001
Total Score Of Cognitive Function(mean [SD])	–	89.641 (23.987)	83.926 (22.896)	87.634 (24.015)	91.684 (23.823)	94.891 (23.775)	<.0001
Diet(mean [SD])	–	49.340 (31.041)	29.951 (27.453)	44.194 (28.082)	55.539 (29.575)	66.182 (26.932)	<.0001
Physical Activity (mean [SD])	–	28.298 (43.031)	7.730 (25.336)	17.993 (36.596)	28.235 (42.561)	56.830 (46.709)	<.0001
Tobacco Exposure (mean [SD])	–	76.700 (31.974)	57.436 (40.433)	77.751 (30.780)	81.755 (26.324)	88.398 (18.802)	<.0001
Sleep Health (mean [SD])	–	82.679 (24.567)	70.822 (29.561)	82.240 (24.107)	86.627 (21.146)	90.193 (18.155)	<.0001
BMI (mean [SD])	–	59.149 (32.724)	41.879 (32.107)	52.814 (31.781)	63.608 (30.164)	6.813 (26.046)	<.0001
Blood Lipids (non-HDL cholesterol) (mean [SD])	–	61.907 (28.888)	48.924 (30.296)	59.176 (27.721)	65.333 (27.348)	73.135 (24.719)	<.0001
Blood_glucose (mean [SD])	–	67.019 (27.973)	51.761 (25.515)	61.452 (27.102)	70.922 (26.270)	82.627 (23.172)	<.0001
BP (mean [SD])	–	47.360 (33.073)	30.861 (28.763)	40.125 (30.371)	53.265 (32.861)	63.923 (30.278)	<.0001
Depression (mean [SD])	–	90.619 (19.330)	89.657 (18.990)	90.412 (19.530)	91.601 (19.069)	90.806 (19.667)	.4274

Mean (SD) for continuous variables, % for categorical variables.

BMI = body mass index, BP = blood pressure, LC9 = life’s crucial 9, NHANES = National Health and Nutrition Examination Survey, PIR = poverty-income ratio, SD = standard deviation.

### 3.2. Association between life’s crucial 9 and cognitive function

Table 2 summarizes the associations between LC9 scores and cognitive function, as evaluated through multivariable linear regression models. We present both unstandardized and standardized coefficients to provide comprehensive insights into the association between LC9 and cognitive function. Table 2 displays the unstandardized results, which show the absolute association in original units, while Table 3 presents standardized coefficients that allow for comparison across different cognitive domains. We have presented the results of both unstandardized and standardized analyses in the main tables (Tables 2–5). In Model 1, which is unadjusted, LC9 scores exhibited a significant positive association with all cognitive measures. These associations remained significant following age adjustment in Model 2. In the fully adjusted Model 3, higher LC9 scores were significantly associated with better performance on all cognitive tests, with consistent results observed for both unstandardized and standardized coefficients. Specifically, for the CERAD total score, a 1-unit increase in LC9 was associated with a 0.03-point increase (β = 0.03, 95% CI: 0.01–0.06, *P* = .006), which corresponds to a 0.38 standard deviation increase (β_std = 0.38, 95% CI: 0.12–0.64). Similarly, for the total cognitive function score, a 1-unit increase in LC9 was associated with a 0.19-point increase (β = 0.19, 95% CI: 0.07–0.32, *P* = .005), equating to a 2.18 standard deviation increase (β_std = 2.18, 95% CI: 0.75–3.61). The standardized coefficients allow for direct comparison of effect sizes across domains, revealing that the association was strongest for the total cognitive function score (β_std = 2.18), followed by the Animal Fluency test (β_std = 0.54), CERAD total recall (β_std = 0.38), and delayed recall (β_std = 0.14). The association with the DSST was of borderline significance in both unstandardized (β = 0.10, *P* = .059) and standardized terms (β_std = 1.12, *P* = .056).

**Table 2:** Association between LC9 and cognitive function (weighted).

	Model 1		Model 2		Model 3	
	β (95% CI)	*P*-value	β (95% CI)	*P*-value	β (95% CI)	*P*-value
CERAD: total score (3 recall trials)	0.06 (0.04–0.08)	<.001	0.06 (0.04–0.08)	<.001	0.03 (0.01–0.06)	.006
CERAD: delayed recall score	0.02 (0.01–0.03)	<.001	0.02 (0.02–0.03)	<.001	0.01 (0.00–0.02)	.024
Animal fluency: total score	0.09 (0.05–0.13)	<.001	0.1 (0.06–0.13)	<.001	0.05 (0.01–0.08)	.012
Digit symbol: score	0.3 (0.15–0.45)	<.001	0.32 (0.19–0.45)	<.001	0.1 (−0.00 to 0.20)	**.059**
Total score of cognitive function	0.47 (0.28–0.66)	<.001	0.5 (0.35–0.66)	<.001	0.19 (0.07–0.32)	.005

Model 1: no covariates were adjusted.

Model 2: age were adjusted.

Model 3: age, gender, race, education level, marital status, PIR, were adjusted.

LC9, Life’s Crucial 9; PIR, Ratio of family income to poverty.

Invalid values are shown in bold.

CI = confidence interval, LC9 = life’s crucial 9.

**Table 3:** Association between LC9 and cognitive function (weighted, β_std).

	Model 1		Model 2		Model 3	
	β (95% CI)	*P*-value	β (95% CI)	*P*-value	β (95% CI)	*P*-value
CERAD: total score (3 recall trials)	0.65 (0.41–0.88)	<.001	0.7 (0.46–0.95)	<.001	0.38 (0.12–0.64)	.006
CERAD: delayed recall score	0.24 (0.14–0.33)	<.001	0.26 (0.17–0.36)	<.001	0.14 (0.02–0.26)	.022
Animal fluency: total score	1.03 (0.59–1.46)	<.001	1.09 (0.72–1.45)	<.001	0.54 (0.13–0.94)	.011
Digit symbol: score	3.36 (1.66–5.06)	<.001	3.61 (2.13–5.09)	<.001	1.12 (−0.03 to 2.28)	**.056**
Total score of cognitive function	5.27 (3.16–7.39)	<.001	5.66 (3.91–7.41)	<.001	2.18 (0.75–3.61)	.005

Model 1: no covariates were adjusted.

Model 2: age were adjusted.

Model 3: age, gender, race, education level, marital status, PIR, were adjusted.

LC9, Life’s Crucial 9; PIR, Ratio of family income to poverty.

Invalid values are shown in bold.

CI = confidence interval, LC9 = life’s crucial 9, PIR = poverty-income ratio.

**Table 4:** Association between LC9 components and cognitive function (weighted).

	Model 1		Model 2		Model 3	
	β (95% CI)	*P*-value	β (95% CI)	*P*-value	β (95% CI)	*P*-value
Diet	0.11 (0.06–0.17)	<.001	0.15 (0.10–0.20)	<.001	0.07 (0.02–0.11)	.004
Physical activity	0.07 (0.03–0.11)	.002	0.05 (0.01–0.09)	.016	0.02 (−0.01 to 0.05)	**.109**
Tobacco exposure	0.05 (0.00–0.11)	**.05**	0.1 (0.05–0.14)	<.001	0.02 (−0.02 to 0.06)	**.269**
Sleep health	0.13 (0.07–0.20)	<.001	0.14 (0.08–0.20)	<.001	0.03 (−0.01 to 0.07)	**.086**
BMI	0.01 (−0.05 to 0.06)	**.845**	0.06 (0.01–0.11)	.029	0.02 (−0.02 to 0.05)	**.313**
Blood Lipids (non-HDL cholesterol)	−0.07 (−0.12 to −0.03)	.003	−0.05 (−0.09 to −0.00)	.045	−0.03 (−0.07 to 0.01)	**.103**
Blood_glucose	0.17 (0.08–0.26)	<.001	0.16 (0.09–0.23)	<.001	0.06 (0.01–0.11)	.02
BP	0.14 (0.09–0.18)	<.001	0.09 (0.05–0.13)	<.001	0.06 (0.02–0.09)	.002
Depression	0.19 (0.09–0.29)	<.001	0.22 (0.11–0.33)	<.001	0.13 (0.05–0.21)	.004

Model 1: no covariates were adjusted.

Model 2: age were adjusted.

Model 3: age, gender, race, education level, marital status, PIR, were adjusted.

Invalid values are shown in bold.

BMI = body mass index, BP = blood pressure, CI = confidence interval, LC9 = life’s crucial 9, PIR = poverty-income ratio.

**Table 5:** Association between LC9 components and cognitive function (weighted, β_std).

	Model 1		Model 2		Model 3	
	β (95% CI)	*P*-value	β (95% CI)	*P*-value	β (95% CI)	*P*-value
Diet	3.55 (1.72–5.37)	<.001	4.68 (3.14–6.22)	<.001	2.1 (0.76–3.43)	.004
Physical activity	3.06 (1.21–4.91)	.002	2.04 (0.41–3.68)	.016	1.08 (−0.20 to 2.35)	**.093**
Tobacco exposure	1.71 (0.00–3.41)	**.05**	3.07 (1.59–4.56)	<.001	0.72 (−0.62 to 2.06)	**.278**
Sleep health	3.27 (1.62–4.92)	<.001	3.45 (1.91–4.99)	<.001	0.83 (−0.15 to 1.81)	**.093**
BMI	0.18 (−1.67 to 2.03)	**.845**	1.88 (0.20–3.55)	.029	0.54 (−0.60 to 1.68)	**.335**
Blood Lipids (non-HDL cholesterol)	−2.13 (−3.45 to −0.79)	.003	−1.32 (−2.61 to −0.03)	.045	−0.88 (−1.95 to 0.18)	**.099**
Blood_glucose	4.74 (2.34–7.14)	<.001	4.55 (2.54–6.56)	<.001	1.63 (0.26–3.00)	.022
BP	4.53 (3.10–5.97)	<.001	3.01 (1.70–4.33)	<.001	1.89 (0.78–3.01)	.002
Depression	3.7 (1.75–5.65)	<.001	4.19 (2.10–6.29)	<.001	2.5 (0.89–4.10)	.004

Model 1: no covariates were adjusted.

Model 2: age were adjusted.

Model 3: age, gender, race, education level, marital status, PIR, were adjusted.

Invalid values are shown in bold.

BMI = body mass index, BP = blood pressure, CI = confidence interval, LC9 = life’s crucial 9, PIR = poverty-income ratio.

### 3.3. Association between life’s crucial 9 components and cognitive function

**Figure 2.**
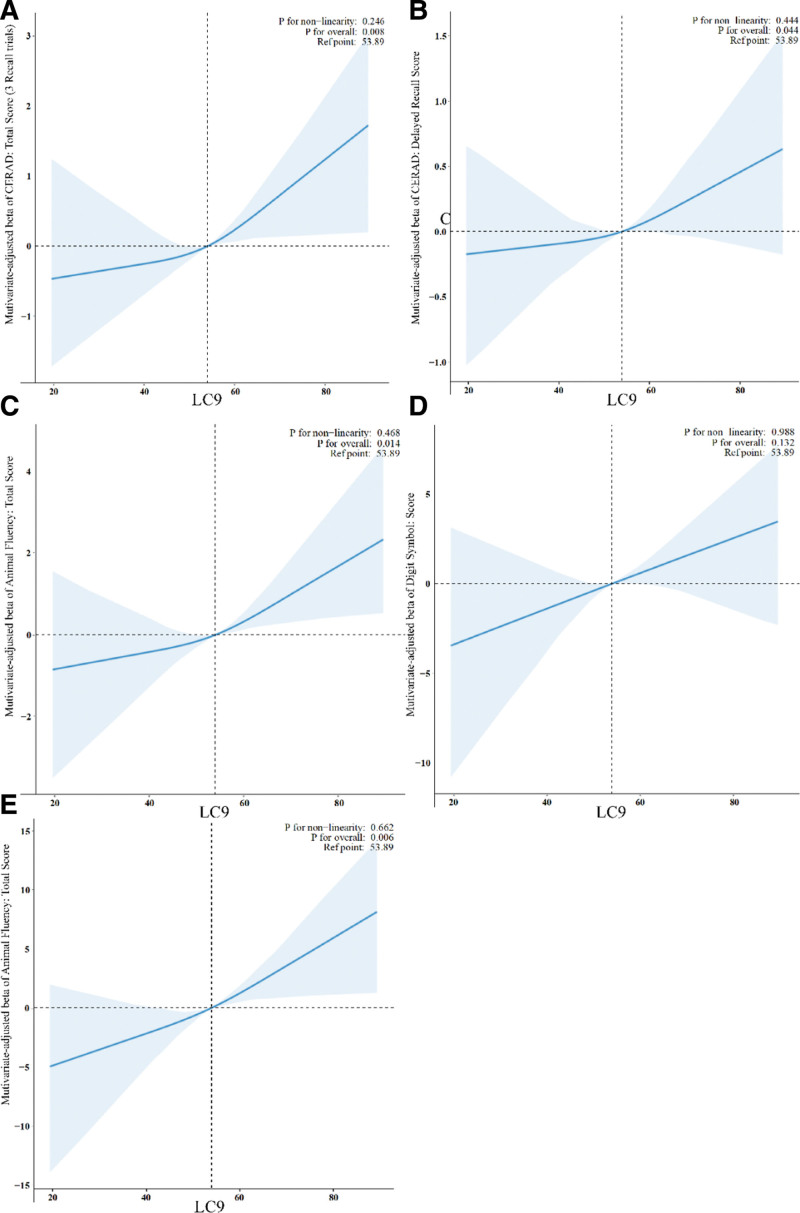
Dose-response relationship between LC9 scores and total cognitive function scores. RCS analysis illustrating the association between LC9 scores and total cognitive function scores in fully adjusted models. The solid line represents the estimated β coefficient, and the shaded area represents the 95% confidence interval. Models adjusted for age, sex, race/ethnicity, education, marital status, and poverty-income ratio. LC9 = life’s crucial 9, RCS = restricted cubic spline.

The associations between individual LC9 components and the global cognitive function score are detailed in Table 4 (unstandardized β) and Table 5 (standardized β_std). In the unadjusted Model 1, significant associations were observed for diet (β = 0.11, 95% CI: 0.06–0.17, *P* <.001, β_std = 3.55, 95% CI: 1.72–5.37, *P* <.001,), sleep health (β = 0.13, 95% CI: 0.07–0.20, *P* <.001, β_std = 3.27, 95% CI: 1.62–4.92, *P* <.001), BG (β = 0.17, 95% CI: 0.08–0.26, *P* <.001, β_std = 4.74, 95% CI: 2.34–7.14, *P* <.001), BP (β = 0.14, 95% CI: 0.09–0.18, *P* <.001, β_std = 4.53, 95% CI: 3.10–5.97, *P* <.001), and depression (β = 0.19, 95% CI: 0.09–0.29, *P* <.001, β_std = 3.7, 95% CI:1.755.65, *P* <.001) with cognitive function. Upon adjusting for age in Model 2, diet (β = 0.15, β_std = 4.68, *P* <.001), sleep health (β = 0.14, β_std = 3.45, *P* <.001), BG (β = 0.16, β_std = 4.55, *P* <.001), BP (β = 0.09, β_std = 3.01, *P* <.001), and depression (β = 0.22, β_std = 4.19, *P* <.001) remained significant in the fully adjusted Model 3, several components remained significantly associated with the global cognitive function score. For instance, a 1-point increase in the Diet score was associated with a 0.07-point increase in the cognitive function score (β = 0.07, 95% CI: 0.02–0.11, *P* = .004). When expressed in standardized terms, this corresponds to a 2.10 standard deviation increase in the cognitive score per 1-SD increase in the Diet score (β_std = 2.10, 95% CI: 0.76–3.43). Similarly, a 1-point increase in the DEPRESSION score was associated with a 0.13-point increase in the cognitive score (β = 0.13, 95% CI: 0.05–0.21, *P* = .004), which had the largest effect size in standardized terms (β_std = 2.50, 95% CI: 0.89–4.10)The standardized coefficients revealed that Depression (β_std = 2.50, 95% CI: 0.89–4.10), Diet (β_std = 2.10, 95% CI: 0.76–3.43), and BP (β_std = 1.89, 95% CI: 0.78–3.01) had the strongest associations with global cognitive function, allowing for a clear ranking of component importance that is not readily apparent from the unstandardized coefficients alone. Other components, such as Physical Activity (β_std = 1.08, *P* = .093), Sleep Health (β_std = 0.83, *P* = .086), and non-HDL Cholesterol (β_std = −0.88, *P* = .099), were not significantly associated with cognitive function in the fully adjusted model based on standardized coefficients, which aligns with the conclusions drawn from the unstandardized results. The dose-response relationship between LC9 scores and cognitive function is depicted in Figure 2, with restricted cubic spline analyses confirming a positive, graded association.

### 3.4. Subgroup analysis

**Figure 3.**
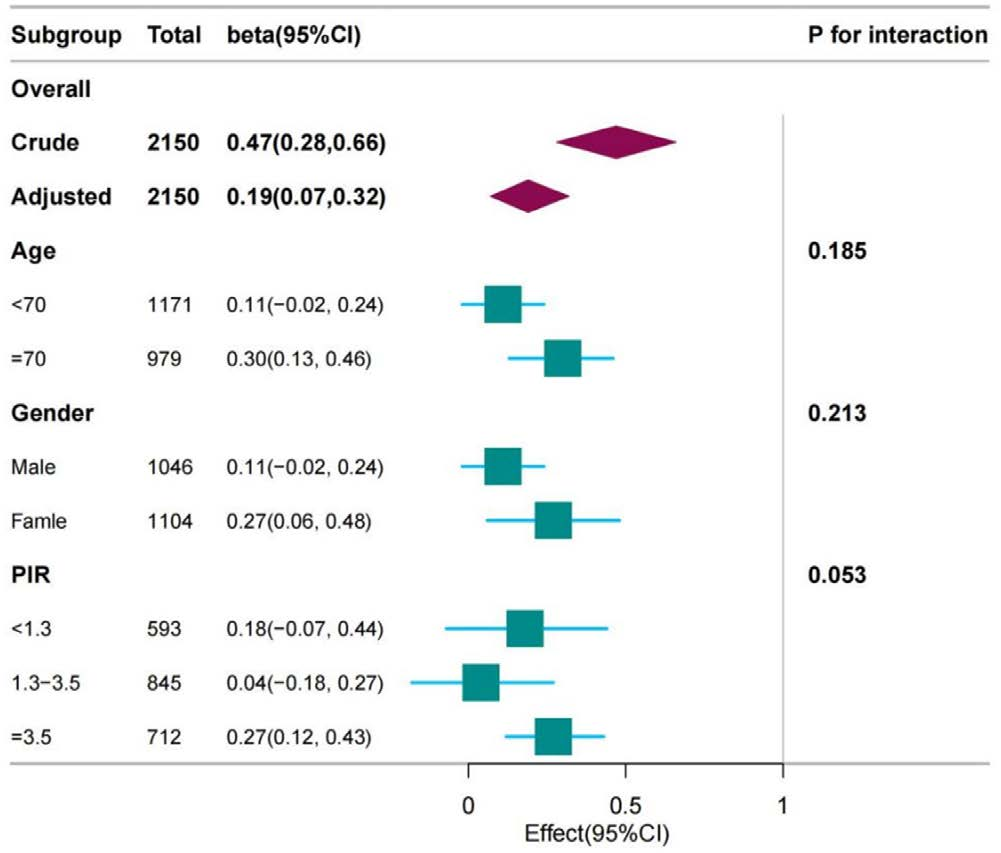
Forest plot of subgroup analyses for the association between LC9 scores and cognitive function. Forest plot presenting β coefficients and 95% confidence intervals for the association between LC9 scores and cognitive function stratified by age group (<70 yr vs ≥70 yr), sex (male vs female), and income level. Analyses conducted using multivariable linear regression, fully adjusted for age, sex, race/ethnicity, education, marital status, and poverty-income ratio. LC9 = life’s crucial 9.

Figure 3 illustrates subgroup analyses examining the relationship between LC9 scores and cognitive function. In the unadjusted model, a strong association was observed (β = 0.47, 95% CI: 0.28–0.66). Following full adjustment, the association remained significant (β = 0.19, 95% CI: 0.07–0.32, p < 0.01). Among participants aged ≥ 70 years, the association was more pronounced (β = 0.30, 95% CI: 0.13–0.46, *P* = .001), whereas the association among those <70 years was not significant (β = 0.11, 95% CI: −0.02–0.24, *P* = .10). Sex-stratified analyses revealed a significant association in women (β = 0.27, 95% CI: 0.06–0.48, *P* = .01) but not in men (β = 0.11, 95% CI: −0.02–0.24, *P* = .08). In income-stratified analyses, significant positive associations between LC9 and cognitive function were observed across all income strata (data not shown). These findings suggest that the beneficial effects of higher LC9 scores on cognitive function may be more pronounced in older adults and women.

## 4. Discussion

This study utilized nationally representative data from the NHANES to investigate the associations between Life’s Crucial 9 (LC9) and cognitive function among U.S. adults aged 60 years and older. A key strength of our analysis is the simultaneous reporting of unstandardized and standardized regression coefficients. The unstandardized coefficients (β) provide directly actionable, clinical insights – for example, quantifying the expected point change in a specific cognitive test per unit improvement in an LC9 component. This is invaluable for clinicians and patients to understand the potential tangible benefits of lifestyle modifications. Conversely, the standardized coefficients (β_std) are indispensable for comparing the relative predictive strength of the LC9 score and its components across the different cognitive domains, which were measured on inherently different scales. For example, our finding that the total cognitive function score (β_std = 2.18) and the depression component (β_std = 2.50) exhibited the largest standardized effects highlights their predominant roles, an insight that might be obscured when only examining the original units of each test. By presenting both metrics, we cater to the needs of both clinical interpretability and statistical comparability, providing a more comprehensive understanding of the relationships between cardiovascular/metabolic health and cognitive performance. Our findings indicate higher LC9 scores are significantly associated with better cognitive function in older U.S. adults, including memory, executive function, and processing speed. These findings align with and extend the growing body of evidence linking modifiable lifestyle and cardiometabolic factors to cognitive health in aging populations, as previously demonstrated in studies on Life’s Essential 8 (LE8) and other multidimensional health indices.^[13,18]^ Three key observations emerge from our analysis.

First, the graded association between elevated LC9 scores and enhanced cognitive outcomes substantiates the hypothesis that the cumulative management of multiple lifestyle and health factors confers additive benefits for cognitive aging. This finding is consistent with the “cognitive reserve” framework, which posits synergistic health behaviors that may bolster neural resilience against age-related decline.^[19,20]^ The persistence of this association after full adjustment underscores the value of integrated, multidomain approaches to preserving cognitive function, echoing findings from prior studies on cardiovascular health metrics.

Second, our component-specific analysis revealed that diet quality, BP, BG, and mental health remained significantly associated with cognitive performance after comprehensive adjustment, suggesting their potential direct neuroprotective roles. These findings align with established neuroprotective pathways: for example, Mediterranean-style dietary patterns may mitigate neuroinflammation and improve metabolic health,^[21]^ while optimal BP and glucose control help preserve cerebrovascular integrity and cerebral perfusion.^[22]^ The significant association with mental health, particularly depression, corroborates previous reports of its independent impact on cognitive trajectories, potentially through reducing glucocorticoid-mediated hippocampal atrophy.^[16]^ Notably, the inclusion of mental health in the LC9 may provide a more holistic assessment of cognitive risk compared to the LE8 framework, addressing an important gap in prior composite metrics. In contrast, the associations of physical activity and BMI were attenuated after accounting for cardiometabolic intermediates, implying their effects may be largely mediated through downstream biological pathways.^[23,24]^

Third, the association between the LC9 and processing speed, as assessed by the DSST, was of borderline significance. This observation aligns with evidence from other lifestyle-cognition studies, including those based on the LE8 framework, which frequently identify processing speed as a domain that is particularly susceptible to age-related decline and may require longer-term or more intensive interventions to demonstrate clear benefits.

LC9 includes 5 health parameters (normal BP, BG, healthy weight, normal cholesterol, physical activity, and sleep) and 4 health behaviors (diet, physical exercise, quitting smoking, and mental health).

Currently, dietary patterns have garnered considerable attention due to their diverse effects on brain health. While earlier research primarily focused on individual nutrients, such as omega-3 fatty acids,^[25]^ contemporary studies underscore the significance of holistic dietary strategies, including the Mediterranean diet, DASH (Dietary Approaches to Stop Hypertension), and MIND (Mediterranean-DASH Intervention for Neurodegenerative Delay). It has been demonstrated that these methods improve cognitive function.^[26,27]^ Notably, the effectiveness of these dietary patterns is attributed to their ability to enhance cognitive performance through several key mechanisms, including anti-inflammatory effects,^[28]^ modulation of the gut-brain axis,^[29]^ and optimization of mitochondrial function.^[30]^ BP and BG levels are important indicators of cognitive health, as evidenced by a growing body of research. Research has shown that increased variability in BP correlates with diminished cognitive function in older adults.^[31]^ Aggressive blood pressure treatment can also significantly decrease cognitive loss in hypertensive adults, according to data from the Systolic BP Intervention Trial (SPRINT) Memory Study.^[32]^ Additionally, comprehensive meta-analyses have shown that both prediabetes and diabetes are linked to a higher risk of dementia and cognitive decline.^[33,34]^ The relationship among BP, BG, and cognitive function is complex and may be mediated through multiple mechanisms, such as oxidative stress,^[35]^ cerebrovascular injury,^[36]^ and neuroinflammation.^[11]^ In recent years, the relationship between mental health issues, especially depression, and cognitive function has been extensively studied. Depression not only affects mood and quality of life but also has the potential to lead to significant declines in cognitive abilities. Studies reveal that people with depression frequently have deficiencies in executive function, memory, attention, and speed of information processing.^[37,38]^ Cognitive impairments are likely linked to several biopsychological mechanisms, including neurobiological changes,^[39]^ inflammatory responses,^[40]^ and cognitive biases.^[41]^ The findings presented herein have significant implications for public health. Promoting optimal cardiometabolic management, supporting mental health, enhancing dietary quality, and paying attention to sleep health may collectively contribute to the preservation of cognitive function in older adults. Integrating LC9-informed strategies into clinical care and community health initiatives could bolster efforts to mitigate the burden of cognitive decline at the population level. The LC9 framework, with its inclusion of mental health, offers a more comprehensive tool for risk stratification and targeted intervention compared to previous models.

This study benefits from several strengths, including the use of a large, nationally representative sample from NHANES, which enhances the generalizability of our findings. Furthermore, our comprehensive assessment of cognitive function through multiple tests provides a detailed understanding of how various lifestyle factors influence cognitive health.

However, there are limitations to consider. Because people may overreport good behaviors or underreport unhealthy ones, biases may be introduced when lifestyle factors are based solely on self-reported data. The cross-sectional design of the study prevents us from demonstrating a causal link between LC9 components and cognitive function. To elucidate the directionality of these correlations and investigate possible mechanisms underlying the observed relationships, further longitudinal research and mechanism research is required.

## 5. Conclusion

In conclusion, this study provides novel evidence suggesting that higher Life’s Crucial 9 (LC9) scores are associated with improved cognitive function among older adults in the United States. These findings underscore the significant public health opportunity and the critical importance of integrating multidomain lifestyle and cardiometabolic interventions into aging-related health policies and programs for promoting cognitive health across the lifespan. Given its strong association with cognitive outcomes and feasibility as a composite metric, the LC9 framework could be considered for potential integration into routine public health screening initiatives to identify individuals at risk for cognitive decline. Future research should prioritize longitudinal validation and interventional studies to further inform the development and policy implementation of effective, evidence-based strategies for enhancing cognitive resilience in aging populations.

## Author contributions

**Conceptualization**: Fan Wang, Hao Liu.

**Data curation**: Xiangyang Wang, Fan Wang, Chaoshuai Hu, Haigang Chang.

**Formal analysis**: Xiangyang Wang, Junwei Wang.

**Funding acquisition**: Haigang Chang.

**Investigation**: Fan Wang, Weiwei Wang, Haigang Chang.

**Methodology**: Xiangyang Wang, Fan Wang, Chaoshuai Hu, Junwei Wang.

**Project administration**: Xiangyang Wang, Weiwei Wang.

**Resources**: Xiangyang Wang, Weiwei Wang.

**Software**: Xiangyang Wang, Weiwei Wang, Hao Liu.

**Supervision**: Junwei Wang, Haigang Chang.

**Validation**: Xiangyang Wang, Fan Wang, Chaoshuai Hu.

**Writing – original draft**: Xiangyang Wang, Fan Wang, Weiwei Wang, Chaoshuai Hu, Junwei Wang, Hao Liu.

**Writing – review & editing**: Haigang Chang.

## Supplementary Material
